# Subclinical Postpartum Renal Structure After Hypertensive Pregnancy Disorders

**DOI:** 10.1161/HYPERTENSIONAHA.125.25130

**Published:** 2025-08-31

**Authors:** Hannah R. Cutler, Jamie Kitt, Prenali D. Sattwika, Lucy E.M. Finnigan, Ana Estevez-Fernandez, Yvonne Kenworthy, Katie Suriano, Annabelle Frost, Samuel Krasner, Casey Johnson, Annabelle McCourt, Rebecca Mills, Katherine Tucker, Alexandra Cairns, Cristian Roman, Christina Aye, Lucy Mackillop, Basky Thilaganathan, Lucy C. Chappell, Betty Raman, Adam J. Lewandowski, Winok Lapidaire, Paul Leeson

**Affiliations:** Cardiovascular Clinical Research Facility, Division of Cardiovascular Medicine, Radcliffe Department of Medicine, University of Oxford, United Kingdom (H.R.C., J.K., P.D.S., Y.K., K.S., A.F., S.K., C.J., A.M., K.T., W.L., P.L.); Nuffield Department of Primary Care Health Sciences, University of Oxford, United Kingdom (J.K.); National Institute for Health Research, Oxford Biomedical Research Centre, Division of Cardiovascular Medicine, Radcliffe Department of Medicine, United Kingdom (L.E.M.F., B.R.); British Heart Foundation Centre for Research Excellence, Radcliffe Department of Medicine, United Kingdom (L.E.M.F., B.R.); Nuffield Department of Women’s and Reproductive Health, University of Oxford, United Kingdom (A.F., K.T., A.C., C.A., L.M.); Oxford Centre for Clinical Magnetic Resonance Research, Division of Cardiovascular Medicine, Radcliffe Department of Medicine, United Kingdom (R.M.); Department of Engineering Science, Institute of Biomedical Engineering (C.R.); Nuffield Department of Population Health, University of Oxford, United Kingdom (A.J.L.); Department of Internal Medicine, Faculty of Medicine, Public Health, and Nursing, Universitas Gadjah Mada, Indonesia (P.D.S.).; Grupo de Investigación Cardiovascular, Departamento de Ciencias de la Salud, Universidade da Coruña, España (A.E.F.).; Fetal Medicine Unit, Oxford University Hospitals National Health Service Foundation Trust, United Kingdom (C.A.).; Fetal Medicine Unit, St George’s University Hospitals National Health Service Foundation Trust, London, United Kingdom (B.T.).; Molecular and Clinical Sciences Research Institute, St George’s, University of London, United Kingdom (B.T.).; King’s College London and Guy’s and St Thomas’ National Health Service Foundation Trust, London, United Kingdom (L.C.C.).; National Institute for Health Care and Research, Oxford Biomedical Research Centre, Oxford, United Kingdom (C.R., P.L.).

**Keywords:** hypertension, kidney, magnetic resonance imaging, postpartum period, pre-eclampsia

## Abstract

**BACKGROUND:**

Hypertensive pregnancies are associated with increased risks of renal failure in pregnancy and later life. However, traditional markers of renal function normalize postpartum, making identification of those at future disease risk difficult. We studied whether the type and severity of hypertensive pregnancy associated with postpartum renal structure.

**METHODS:**

One hundred and twenty-five women from interventional trials (61 preeclamptic, 33 gestational hypertension, and 31 normotensive pregnancy), aged ≥18 years, were imaged using magnetic resonance imaging 6 to 12 months postpartum. Anthropometric, demographic, blood pressure, and blood sample data were collected during pregnancy and postpartum. Kidney volume indexed to body surface area and corticomedullary differentiation were compared between groups using a 1-way ANCOVA, whereas associations with other outcomes were assessed using correlation tests.

**RESULTS:**

Postpartum total kidney volume indexed to body surface area was smaller in women who had preeclampsia compared with those who had gestational hypertension or a normotensive pregnancy (*P*=0.049). Total kidney volume postpartum correlated with estimated glomerular filtration rate at delivery (*P*<0.001). However, smaller volumes were not explained by reduced corticomedullary differentiation, which only differed in women with gestational hypertension compared with preeclamptic (*P*=0.02) and normotensive women (*P*=0.007). There were no associations between renal measures and blood pressure during or after pregnancy.

**CONCLUSIONS:**

At 6 to 12 months postpartum, preeclamptic women have smaller kidney volumes than women with gestational hypertension or normotensive pregnancies. These smaller volumes relate to lower renal function at delivery but not corticomedullary differentiation, which only differed in women with gestational hypertension.

**REGISTRATION:**

URL: https://www.clinicaltrials.gov; Unique identifiers: NCT04273854 and NCT05434195.

**NOVELTY AND RELEVANCE**

**What Is New?**

This is the first study to our knowledge to use magnetic resonance imaging to compare kidney structure in women with hypertensive and normotensive pregnancies 6 to 12 months postpartum.

**What Is Relevant?**

Hypertensive disorders are one of the leading causes of maternal and perinatal deaths globally and are linked to elevated long-term risks of chronic kidney disease and end-stage renal failure. The findings indicate renal structural changes are evident early in life in women with hypertensive pregnancy disorders.

**Clinical/Pathophysiology Implications?**

These findings suggest that kidney volume postpartum is associated with the degree of renal dysfunction during pregnancy. Careful consideration of renal characteristics may add value to the identification of women at increased lifetime risk of pregnancy disorders and later diseases.

Pregnancy places substantial physiological demands on the kidneys, leading to a 30% increase in kidney volume and a 40% to 50% rise in glomerular filtration rate to maintain physiological homeostasis.^1^ In healthy pregnancy, the kidneys adapt effectively to these changes, and renal function remains close to baseline. However, in hypertensive pregnancies, impaired renal function is common, and a third of new acute kidney injuries in pregnancy are in hypertensive women.^2^ These women also face substantially increased lifetime risk of renal disease.^3–9^

Women with preeclampsia, in particular, have a 5-fold increased risk of end-stage renal failure later in life compared with women with normotensive pregnancies.^9^ This heightened risk is not unexpected, given that proteinuria and impaired renal function are hallmark features of preeclampsia.^3–9^ To what extent longer-term risk relates to renal damage during pregnancy, or preexisting renal structural differences that predispose to a decline in renal function in response to the stress of pregnancy, is unclear. However, traditional markers of renal dysfunction, such as proteinuria, decreased glomerular filtration rate, and the acute renal lesions, seen in preeclampsia, typically resolve postpartum.^3^ Therefore, postpartum identification of women most at risk of future renal dysfunction, who may benefit from early preventive strategies, remains difficult.

We hypothesized that imaging might identify postpartum subclinical variations in the renal system in women who have had hypertensive disorders of pregnancy and that these would be most apparent in those who had preeclampsia. Such changes could help define biomarkers to identify women at risk of later renal diseases. In this study, we used magnetic resonance imaging to characterize renal size and microstructural integrity, as a marker of renal damage. Findings were related to renal function during and after pregnancy, type of hypertensive pregnancy disorder, and the impact of postpartum blood pressure management.

## Methods

### Data Availability

Data are available from the chief investigator, P.L., on reasonable request, subject to the approval of the Sponsor, University of Oxford, and the Trial Steering Committees.

### Study Design and Population

A secondary analysis of 2 clinical research studies was performed with detailed demographic, clinical, and pregnancy-related data on women with hypertensive and normotensive pregnancies. The hypertensive cohort (gestational hypertension/preeclampsia) was enrolled in the renal imaging substudy of the POP-HT trial [Physician Optimized Postpartum Hypertension-Treatment Trial]),^10–12^ where they were randomized to telemonitored home blood pressure management or standard National Health Service (NHS) care immediately postpartum. The normotensive cohort was enrolled as a control group in the ongoing CAREFOL-HT study (Clinical Antenatal Randomized Study to Characterize Key Roles of Tetrahydrofolate in Hypertensive Pregnancies).

Participants were recruited between January 2020 and July 2024 from the John Radcliffe Hospital Women’s center (Oxford University Hospitals NHS Foundation Trust), with ethical and research governance approval obtained (POP-HT: 19/LO/1901; CAREFOL-HT: 21/WA/1069). All participants were aged ≥ 18 years and fit the inclusion and exclusion criteria. Information on sex, race, and ethnicity were self-reported in line with the National Institute for Health Research categories. Full details of the methods have been reported previously.^10–12^ Both cohorts had follow-up assessments conducted ≈9 months postpartum (ranging between 6 and 12 months), including identical magnetic resonance imaging protocols and data collection frameworks. Further details regarding the definition of hypertensive groups, inclusion, and exclusion criteria, as well as additional methodological information, are provided in the Supplemental Methods.

### Data Collection

All data were collected at the John Radcliffe Hospital, Oxford, United Kingdom. Antenatal information was collected at the standard 12-week booking appointment. Baseline bloods were collected 1 day post delivery, and postpartum renal assessments were completed between 6 to 12 months postpartum.

### Magnetic Resonance Imaging Acquisition and Analysis

Renal magnetic resonance was performed using a 3.0 Tesla Prisma Scanner (Siemens Medical Solutions, Erlangen, Germany) at the Oxford Center for Clinical Magnetic Resonance Research (John Radcliffe Hospital, Oxford, United Kingdom). An 18-channel phased array surface coil was placed over each patient’s abdomen as they lay in a supine position. Images were captured without contrast. All imaging sequences, scanner settings, and acquisition parameters were consistent across studies.

**Figure 1.**
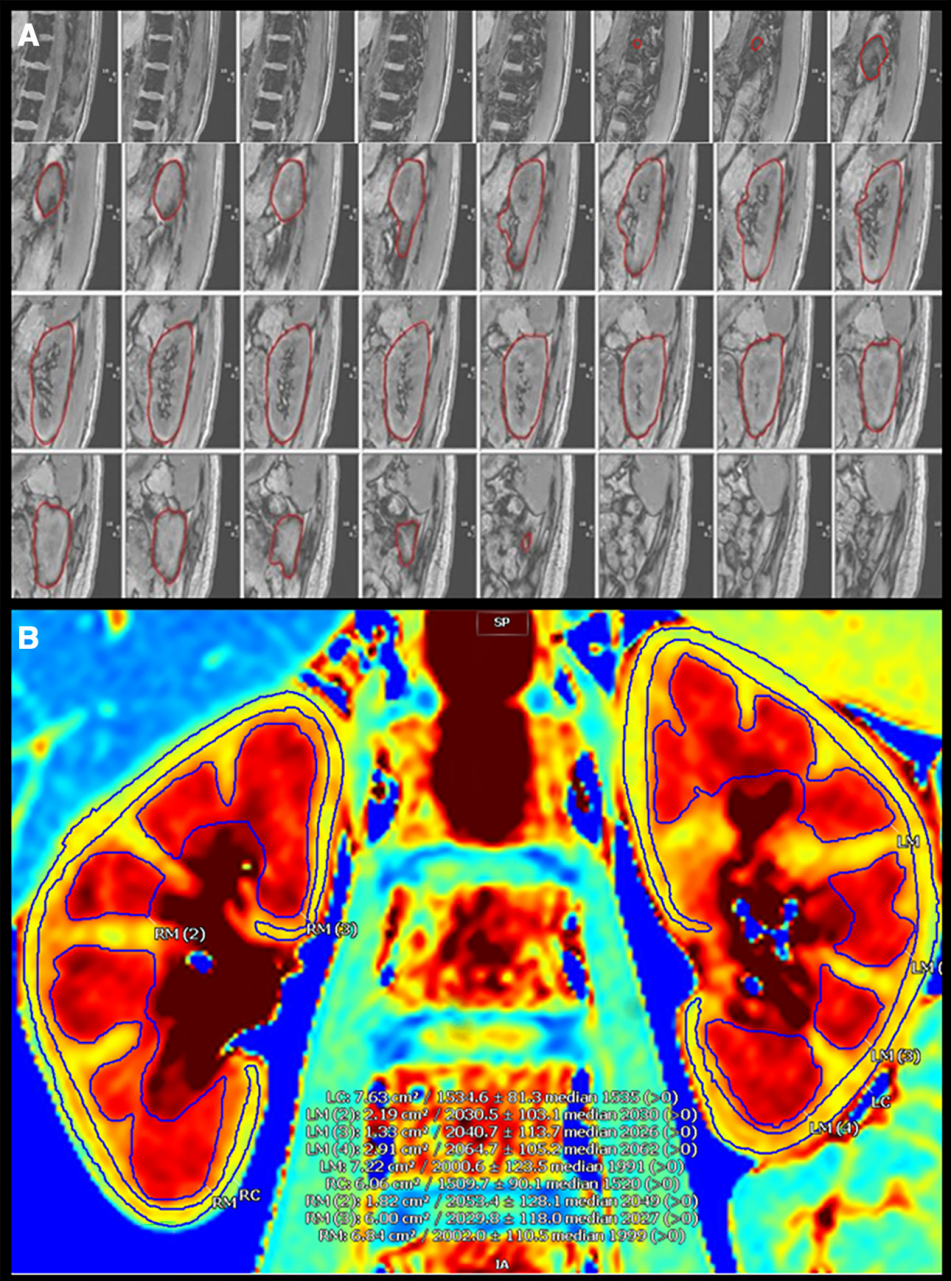
**Analysis of renal structure using magnetic resonance imaging. A**, Kidney volume calculations using total voxel approach. **B**, Corticomedullary differentiation contours. A color scale from 50 to 2500 arbitrary units (a.b.u) was applied to enhance the contrast between the renal cortex and medulla and to help assess the integrity of kidney structure—2000 (red) was used to identify the medulla and 1500 (yellow) was used to identify the cortex. LC indicates left cortex; LM, left medulla; RC, right cortex; RM, right medulla; and SP, spine.

Axial, coronal, and sagittal localizer scans were collected along with unenhanced 3-dimensional sagittal T2-weighted volumetric interpolated breath-hold images to assess kidney volumes. Volumes were calculated using a voxel-count approach in Cardiovascular Imaging 42 software (Circle, Calgary, Alberta, Canada). Volume analysis for both studies was completed at the same time point by 1 investigator (H.R.C.). This investigator identified and defined organ boundaries on consecutive sagittal slices before manually contouring kidney perimeters (Figure 1A). The software then interpolated between traced slices and summed the voxels to calculate kidney volume. Another blinded investigator (B.R.) verified accuracy.

Total kidney volume was calculated by summing the volumes of both kidneys, which were then indexed to body surface area using the Mosteller formula.^13^ Two researchers (H.R.C. and A.E.F.) reviewed the kidney imaging data to ensure its completeness and quality. During this process, they specifically looked for kidneys that had incomplete image sets—meaning ≥1 image slices were missing. If a kidney scan was missing slices, it was excluded from the analysis to maintain data accuracy and reliability. Interobserver reliability was assessed by comparing the degree of consistency between the first investigator and another blinded investigator (A.E.F) in 20 kidney volumes (left and right) from 10 randomly selected patients across all groups. Intraobserver reliability was assessed by the first investigator (H.R.C.) recontouring 20 randomly selected kidney volumes from 10 patients and comparing the volumes at 2 time points a couple of months apart.

Corticomedullary differentiation, defined as the structural distinction between the cortex and the medulla of the kidney, was calculated using T1 maps from Modified Look-Locker Inversion data.^14^ T1 mapping was computed using a 3-parameter curve fitting of the data to A–B exp (–*x*/T·1) using the Levenberg–Marquardt algorithm, and *R*_2_ and residual maps for quality control. The kidney was segmented by the T2-weighted scans, and regions of interest for the cortex and medulla were defined based on histogram analysis of the T1 maps, excluding the upper and lower semiquartiles for robustness. One investigator (H.R.C.) placed ellipsoid regions of interest in the cortex and medulla (Figure 1B), which were checked by 2 other blinded investigators (B.R. and L.F.).

Mean T1 values for the cortex and medulla were calculated, and corticomedullary differentiation was expressed as a fraction of the cortex divided by the medulla. Renal images were assessed for quality using a standardized 4-point scale, ranging from zero (no artifact) to 3 (major artifact affecting both kidneys). Images receiving a score of 3 were excluded, whereas those with scores of 1 (minor artifact affecting 1 kidney) or 2 (major artifact affecting 1 kidney or minor artifact affecting both kidneys) underwent further review. In cases where artifacts were determined to compromise the reliability of T1 measurements, the affected kidneys were excluded based on predefined criteria applied by the primary reviewer (H.R.C.).

### Other Assessments

Height, weight, hip, waist, and left arm circumference were measured using standardized calibrated equipment. Clinical blood pressures were measured using an automated blood pressure machine (GE Dinamap Carescape V100, United Kingdom) with an appropriately sized cuff placed at least 2.5 cm above the elbow. Three measurements were taken 1 minute apart, and the final 2 measurements were averaged. Venous blood samples were collected via venipuncture. For the hypertensive cohort, samples were processed immediately, but for the normotensive cohort, samples were frozen whole at –80 °C and processed at a later time. Samples from both groups were analyzed by the biochemistry department at the John Radcliffe Hospital. Estimated glomerular filtration rates (eGFRs) for both groups were calculated using the 2021 Chronic Kidney Disease Epidemiology Collaboration equation.^15^

### Statistical Analysis

The primary outcome was the difference in total kidney volume indexed to body surface area across groups. Secondary outcomes include group differences in right and left kidney volumes (accounting for their anatomic position), corticomedullary differentiation on T1 mapping, and blood biomarkers.

The original trials included in this article were conducted according to predefined protocols. However, the specific comparative analyses presented here are exploratory and were not preregistered in a dedicated analysis plan. Nevertheless, comparisons of primary and secondary outcomes across the 3 groups were planned before statistical analysis.

Statistical analysis was conducted using R (version 4.4.2) and R Studio (version 2024.12.0 + 467). Variables were tested for normality using the Shapiro-Wilks test for continuous variables where n ≤ 50 and visual observation using Q-Q plots and histograms for larger samples. Normally distributed continuous variables were reported as means and standard deviations. Skewed continuous variables were reported as medians and interquartile ranges. Categorical data were presented as counts and percentages.

Differences across group means of normally distributed continuous variables were tested using a 1-way ANOVA after the Levene test was performed to check homogeneity of variance. For multiple comparisons, between-group differences were tested using the Tukey Honest Significant Difference test. For variables with skewed distributions, differences across group medians were tested using the Kruskal-Wallis test, with between-group differences tested using the Dunn’s post hoc test and Bonferroni correction for multiple comparisons. Differences across categorical variables were tested using the *χ*^2^ test and post hoc pairwise Z tests. Sensitivity analyses were conducted using ANCOVA, adjusting for time postpartum and age as covariate to assess for any confounding effects.

Correlations were evaluated using Pearson Correlation Coefficient for normally distributed variables and Spearman Rank Correlation Coefficient for variables with skewed distributions. A sensitivity analysis was conducted to investigate the effect of telemonitored home blood pressure monitoring postpartum compared with standard NHS care on renal outcomes in the hypertensive group. This involved a Welch 2-sample *t* test to assess differences in mean total kidney volume between participants who received the intervention and those who did not. The Mann-Whitney *U* test was used to compare corticomedullary differentiation across the 2 groups. In addition, differences in kidney volume and corticomedullary differentiation were compared across early- and late-onset preeclamptic groups using the Mann-Whitney *U* test. Bland-Altman analysis was used to assess interobserver and intraobserver variability (Figures S1 and S2).

## Results

### Study Population

Renal data were available for 125 women who had undergone imaging at 6 to 12 months postpartum (Figure S3). This data set included 61 preeclamptic women and 33 gestational hypertension who had taken part in the renal imaging substudy of the POP-HT trial, as well as 31 women from the normotensive comparator group of the CAREFOL-HT study. Of the preeclamptic group 36 of 61 women (59%) were randomized to the telemonitored home blood pressure management postpartum, and 25 of 61 (41%) were given standard NHS care. Of the gestational hypertension group, 19 (57.6%) were randomized to the telemonitored home blood pressure management postpartum and 14 (42.4%) were given standard NHS care.

Full demographic, anthropometry, and pregnancy characteristics of the renal imaging subgroup are reported in Table 1. Time of follow-up postpartum in days was equivalent in each group as was participant age, but those with normal blood pressure throughout pregnancy had lower body mass index, low-density lipoprotein, triglyceride levels, and blood pressure at 6 to 12 months compared with those with either preeclampsia or gestational hypertension (*P*<0.05).

**Table 1.**
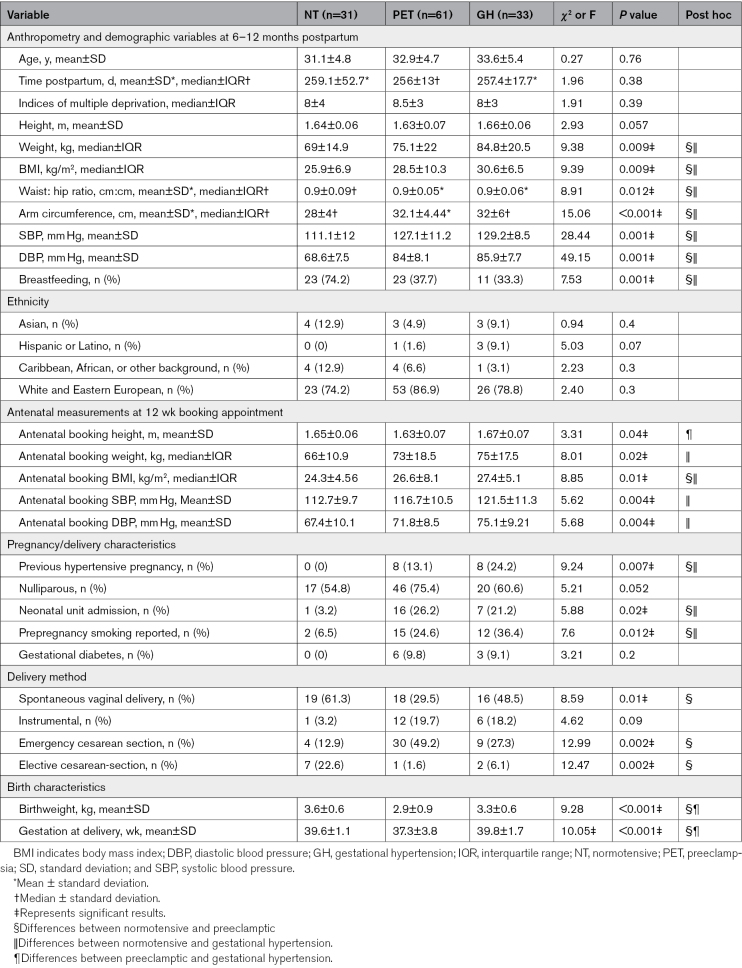
Participant Anthropometry, Demographic Data, Clinical Blood Pressure Readings, Blood Results, and Pregnancy Characteristics

### Kidney Volumes Indexed to Body Surface Area

**Figure 2.**
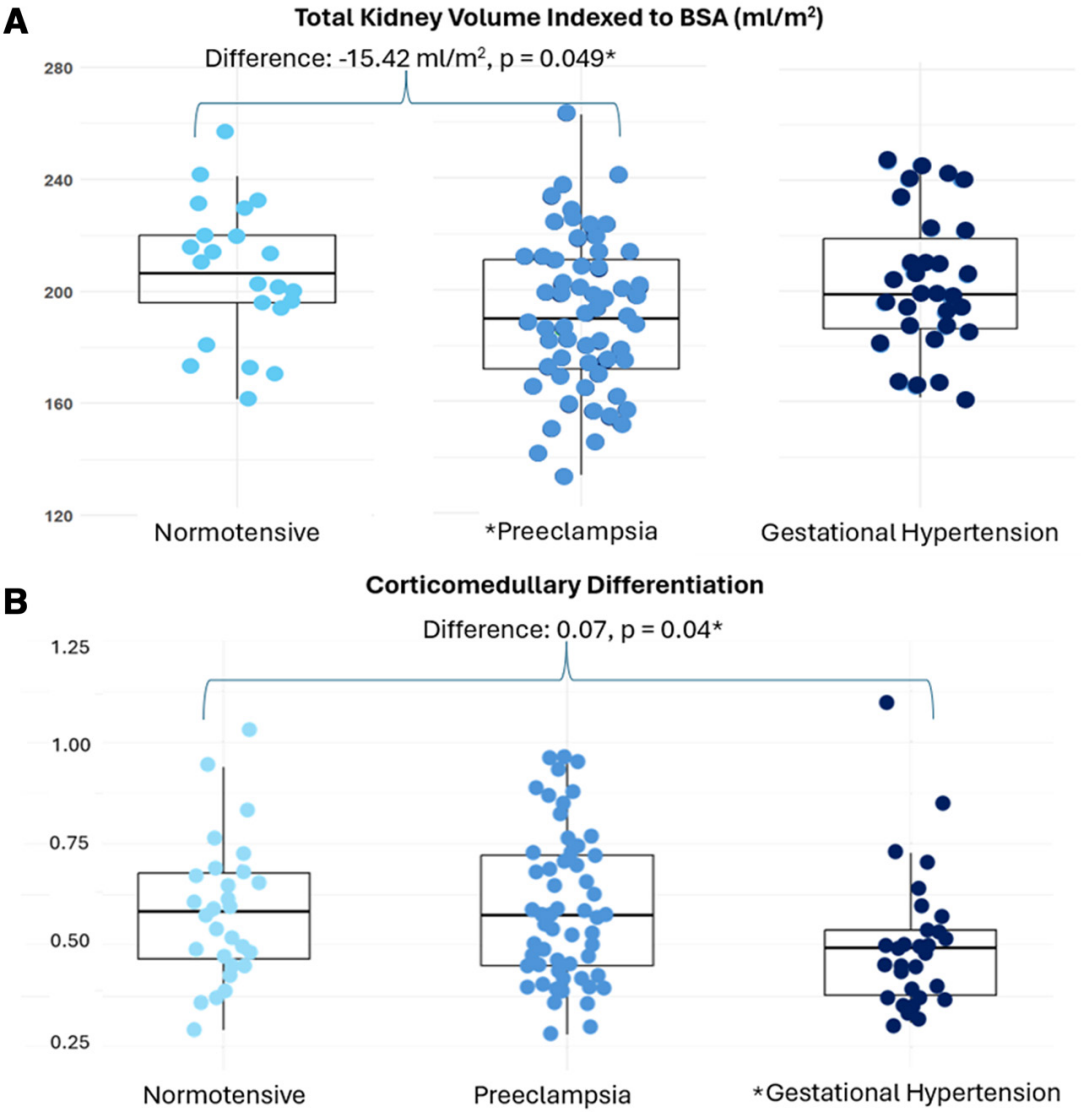
**Boxplots comparing kidney structure across groups. A**, Total kidney volume indexed to body surface area (BSA) across normotensive (light blue), preeclampsia (medium blue), and gestational hypertension (dark blue) groups. *Represents significant results between the normotensive and preeclamptic group. **B**, Corticomedullary differentiation across normotensive (light blue), preeclampsia (medium blue), and gestational hypertension (dark blue) groups. *Represents significant results between the normotensive and gestational hypertension group.

Of the 125 renal imaging data sets, comprising 250 kidneys, 122 right kidney volumes, and 114 left kidney volumes were of sufficient quality for analysis. As a result, total kidney volume could be compared across 114 participants. Total kidney volumes indexed to body surface area varied across the 3 groups (*P*=0.012). After adjusting for age and time postpartum, the group differences remained significant (*P*=0.027), and neither age nor time were significant covariates in the model (*P*=0.46, *P*=0.22, respectively). The difference lay between the preeclamptic and normotensive groups, with the preeclamptic group having lower total volume (mean difference, −15.42 mL/m^2^ [95% CI, −30.84 to −0.005]; *P*=0.049). There were no significant differences between the normotensive pregnancy group and the gestational hypertension group (mean difference, −3.3 mL/m^2^ [95% CI, −20.70 to 14.10]; *P*=0.89; Figure 2).

There was a significant difference in right kidney volume across the groups (*P*=0.02), and a trend towards reduced left kidney volume (*P*=0.09). After adjusting for age and time postpartum, the difference in right kidney volumes remained significant (*P*=0.011), and both age (*P*=0.56) and time postpartum were not significant predictors (*P*=0.12). The preeclamptic group had lower right kidney volumes than the normotensive pregnancy group (mean difference, −7.11 mL/m^2^ [95% CI, [−14.13 to −0.09]; *P*=0.046) and the gestational hypertension group (mean difference, 7.66 mL/m^2^ [95% CI, 0.56−14.76]; *P*=0.03), with no differences between the normotensive and gestational hypertension groups (mean difference, 0.55 mL/m^2^ [95% CI, −7.60 to 8.70]; *P*=0.99).

### Corticomedullary Differentiation

Overall, 115 out of 125 women (92%) had sufficient quality T1 maps for analysis of corticomedullary differentiation. There were no significant differences in mean T1 cortex or medulla between groups (Table 2). However, there was a difference in overall corticomedullary differentiation across the 3 groups (*P*=0.04; Figure 2) due to a reduced corticomedullary differentiation in the gestational hypertension group compared with the preeclamptic group (mean difference, 0.08; *P*=0.02) and the normotensive group (mean difference, 0.07; *P*=0.007). There was no significant effect of age (*P*=0.61) or time postpartum (*P*=0.32) on corticomedullary differentiation.

**Table 2.**
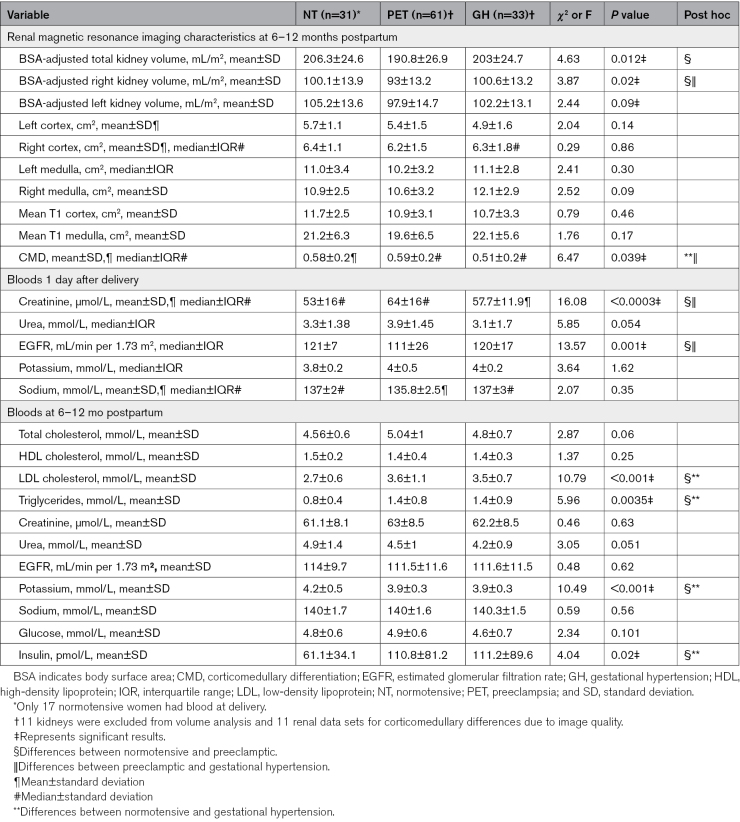
Renal Characteristics at Birth and 6 to 12 Months Postpartum

### Renal Differences in Early Versus Late-Onset Preeclampsia

Out of the 61 preeclamptic women, 13 were classed as early-onset (≤33+6 weeks gestation), and 48 were classed as late-onset (>34 weeks gestation). The early-onset group had on average, lower renal volumes, but there were no significant differences between groups (median difference, 13.3 mL/m^2^; *P*=0.896). The early-onset preeclamptic group also on average had lower corticomedullary differentiation (median difference, 0.077), but this did not reach statistical significance (*P*=0.3683).

### Renal Function

**Figure 3.**
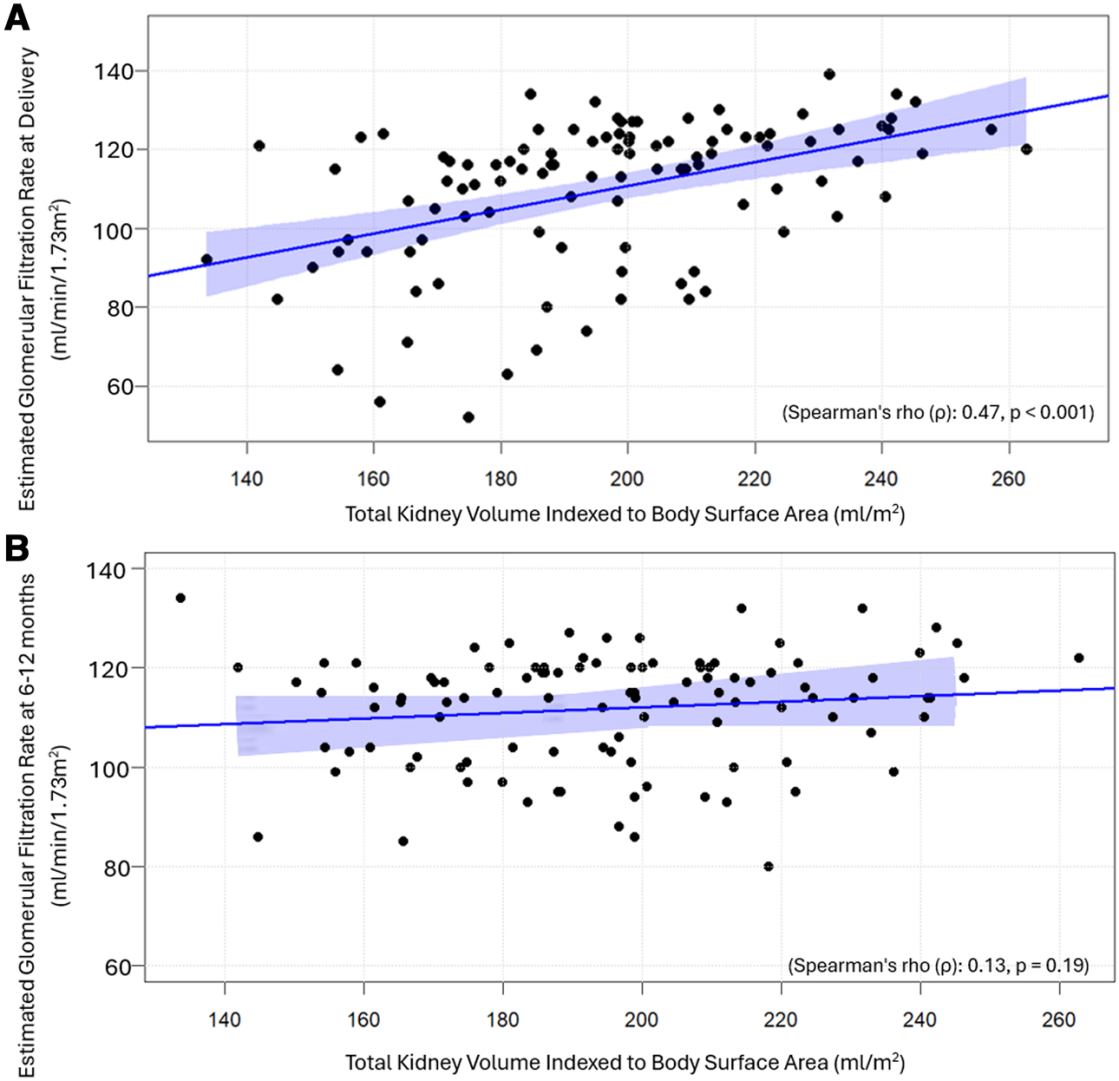
**Linear Regression models showing total kidney volume indexed to body surface area vs estimated glomerular filtration rates at 1 day after delivery and 6 to 12 months postpartum for all participants. A**, Shows the linear regression model at 1 day after delivery. **B**, Shows the linear regression model at 6 to 12 months postpartum.

At time of delivery, 14 out of 61 (23%) women with preeclampsia had eGFR ≤ 90 mL/min per 1.73 m^2^, compared with 3 out of 33 (9%) women with gestational hypertension and 1 out of 17 (5%) women who had a normotensive pregnancy. By the time of postpartum follow-up, eGFRs had returned to >90 mL/min per 1.73 m^2^ for 113 of 119 (94.9%) women. However, they were still ≤90 mL/min per 1.73 m^2^ in 5 women who had preeclampsia and 1 woman with prior gestational hypertension. There were no between-group differences in absolute levels of eGFR or creatinine at time of postpartum follow-up but there were differences at delivery (Table 2). There was a positive correlation between total kidney volume indexed to body surface area at 6 to 12 months postpartum and eGFR 1 day after delivery (Spearman *ρ*=0.47, *P*<0.001) but not with eGFR at 6 to 12 months postpartum (Spearman *ρ*=0.13, *P*=0.19; Figure 3).

### Associations Between Renal Outcomes, Blood Pressure, Body Mass Index, and Blood Pressure Management Postpartum

Kidney volumes indexed to BSA at 6 to 12 months postpartum were not related to systolic (*r*=0.01, *t* [111]=0.11 [95% CI, −0.17 to 0.20]; *P*=0.91) or diastolic blood pressure (*r*=−0.07, *t* [111]=−0.77 [95% CI, −0.25 to 0.12]; *P*=0.44; Figure S4), nor measures of body mass index at the postpartum visit (*P*>0.05).

There was also no association between corticomedullary differentiation, renal function, blood pressure, or body mass index (*P*>0.05). In addition, there was no significant difference between those who had telemonitored home blood pressure management or standard NHS care postpartum in the hypertensive groups on renal outcomes including kidney volume, corticomedullary differentiation, or renal function at 6 to 12 months postpartum (*P*>0.05; Figures S5 and S6).

## Discussion

This study has identified differences in kidney volume and corticomedullary differentiation at 6 to 12 months postpartum in women who had a hypertensive pregnancy compared with women who had a normotensive pregnancy. Women who develop preeclampsia have evidence of reduced kidney volumes, with a graded association between the degree of reduction and the degree of renal filtration capacity evident at delivery. However, the smaller kidney volume was not related to reduced corticomedullary differentiation, which was only evident in those who had gestational hypertension. These findings suggest that there may be different renal pathological pathways underlying the risks of gestational hypertension and preeclampsia.

Previously, a 10 mL reduction in total kidney volume has been associated with a 6% to 10% higher risk of chronic kidney disease later in life.^16^ The difference in kidney volume we observed, which equates to around 24 mL in an average woman, therefore, is significant and would be consistent with the known increase in risk of renal disease for women who have preeclampsia.^3–9^ The presence of these kidney volume differences soon after pregnancy suggests that the substantially higher risk of later renal disease seen in women who have preeclampsia may not be entirely secondary to their known hypertensive and cardiometabolic risks^17^ but may partly be explained by underlying differences in preexisting renal structure or accumulated renal damage during pregnancy.

Differences in kidney volume and impaired corticomedullary differentiation may also signify microvascular injury or maladaptive vascular responses that predispose individuals to hypertension, heart failure, and atherosclerosis. Notably, a UK study involving over 2.8 million women showed that women with hypertensive disorders of pregnancy have a 1.25- to 2-fold higher risk of stroke, acute coronary syndrome, and chronic kidney disease, with even greater risks for heart failure and arrhythmias, especially in cases of recurrent or early-onset preeclampsia.^18^ Although these observations highlight the importance of monitoring this high-risk population, it remains unclear whether imaging differences directly correlate with long-term declines in kidney function or progression to end-stage renal disease.

Longitudinal follow-up studies are needed to clarify whether postpartum renal imaging can effectively identify women with hypertensive pregnancies at increased risk of future renal disease. Although magnetic resonance imaging is unlikely to be cost-effective for routine screening, renal ultrasound could offer a more practical option for high-risk groups such as women with severe preeclampsia. In addition, combining imaging with emerging plasma and urine biomarker panels may provide a more comprehensive approach to understanding disease trajectories and stratifying long-term renal risk.^19^

Interestingly, kidney volume 6 to 12 months postpartum was associated in a graded manner with eGFR around the time of delivery, suggesting there is an association between renal size and the response of the renal system to the stress of pregnancy. Notably, a high proportion of those with preeclampsia had an eGFR ≤ 90 mL/min per 1.73 m^2^ at this time, despite the known rise in eGFR during pregnancy.^9^ By 6 to 12 months postpartum, renal function was similar between groups, and there was no association with blood pressure at booking, during pregnancy, or at follow-up. There was also no impact of postpartum blood pressure self-management on renal measures at follow-up in those who participated in the POP-HT study.^10–12^

Furthermore, no differences in corticomedullary differentiation were evident in those who developed preeclampsia, in whom specific known glomerular changes during pregnancy could have contributed to postpartum kidney volume. This raises the possibility that the differences in kidney volume in those who developed preeclampsia predate the hypertensive pregnancy and the postpartum period. As such, kidney volume may be a marker of risk for decline in renal filtration during a hypertensive pregnancy, and, therefore, risk of progression to preeclampsia. However, as prepregnancy imaging data were not collected in this study. We cannot determine whether the structural kidney differences observed postpartum reflect preexisting susceptibility, injury incurred during pregnancy, or impaired recovery after delivery. Future studies investigating prepregnancy renal structure would be useful to clarify this.

The difference in corticomedullary differentiation exclusively in those with gestational hypertension was unexpected. This diminished distinction between the cortex and medulla may be an early indicator of kidney dysfunction^20^ and can be associated with glomerular damage, tubular injury, or impaired renal blood flow due to sustained high blood pressure for prolonged time periods.^21^ It is possible that this reflects a longer history of hypertensive disease, or additional metabolic or physiological factors associated with a gestational hypertensive phenotype.^22^ It is notable that in this study, women with gestational hypertension were more likely to have a history of hypertensive pregnancy and to smoke, both of which may influence corticomedullary differentiation and warrant consideration as potential contributing factors.

However, women with gestational hypertension also delivered at a later gestational age than those with preeclampsia and the mean antenatal blood pressure at the 12-week booking appointment in the gestational hypertensive group was significantly higher compared with the normotensive group, being 121 ± 11 mm Hg compared with 113 ± 10 mm Hg, with this difference not being evident in those who developed preeclampsia (Table 1). These observations raise the possibility that women who develop gestational hypertension have longer exposure to hypertension or that there are distinct renal pathological pathways underlying these 2 hypertensive pregnancy disorders.

Finally, it is important to note that although we did not find any significant differences between the early and late-onset preeclamptic groups, this may be a result of the small number of early-onset cases in this cohort. Future studies with larger sample sizes could investigate the difference between these groups further.

### Limitations

First, we were unable to obtain blood samples for all the normotensive participants at delivery, as this is not part of standard routine care for this population, and at 6 to 12 months postpartum, 6 women declined having blood taken. Nevertheless, there was no clear selection bias in the characteristics of those participants who provided a sample.

Second, 4.4% of the scans were excluded due to poor quality data, and magnetic resonance imaging was added as a protocol amendment during the POP-HT trial. As a result, participants enrolled in the earlier part of the study were not offered a magnetic resonance imaging scan. This limited the study sample size and may have introduced some selection bias.

However, because recruitment into the substudy was sequential once the amendment was approved, it is unlikely that there could have been any systematic bias. In addition, there was no systematic difference in exclusion rates across groups, suggesting that factors such as body mass index did not introduce bias in corticomedullary or volume assessment.

Third, the generalizability of this study is limited by the relatively homogenous ethnicity, sociodemographic profile, and single-site study design. Kidney disease health disparities are well-recognized, with Black and Hispanic individuals having a 3.4-fold and 1.3-fold greater life risk of developing end-stage renal disease than their white counterparts.^23^ Multicenter studies or more diverse populations could validate and expand the findings. Finally, women with known preexisting hypertension or renal disease were excluded from the study, and further work is needed to determine whether associations are similar in those with preexisting cardiometabolic and cardiorenal disease.

### Conclusions

Women who develop preeclampsia show reduced kidney volume 6 to 12 months postpartum compared with women who had gestational hypertension or normotensive pregnancies. Volume reduction correlates with renal function decline at delivery but is independent of antenatal or postpartum blood pressure, as well as postpartum renal function and corticomedullary differentiation. Women with prior gestational hypertension show differences in corticomedullary differentiation. Further studies are required to understand whether renal biomarkers at delivery, or postpartum kidney volume, may help to identify those at increased risk for renal disease in later life or future hypertensive disorders of pregnancy.

### Perspectives

These results highlight a role of renal structure in the pathophysiology of hypertensive pregnancy disorders. Further research is needed to determine whether subclinical differences in kidney volume and microstructure can identify women who are predisposed to progressing to longer-term renal and cardiovascular disease after hypertensive pregnancy.

## ARTICLE INFORMATION

### Acknowledgments

The authors acknowledge J. Kitt, A.J. Lewandowski, Richard McManus, and P. Leeson, who secured funding for the studies.

### Author Contributions

JJ. Kitt, A. J. Lewandowski, K. Tucker, A. Cairns, K. Suriano, C. Aye, A. Frost, S. Krasner, W. Lapidaire, C. Roman, L. Mackillop, L. C. Chappell, B. Thilaganathan, Y. Kenworthy contributed to the design of POP-HT (Physician Optimized Postpartum Hypertension-Treatment Trial) or CAREFOL-HT (Clinical Antenatal Randomised Study to characterize Key Roles of Tetrahydrofolate in Hypertensive Pregnancies). H.R. Cutler, J. Kitt, A.J. Lewandowski, P. Leeson, and W. Lapidaire contributed to the design of the follow-up visit. P. Leeson, W. Lapidaire, K. Suriano, and A.J. Lewandowski refined the overall study protocol, led project delivery, and provided guidance and external refinement. A.J. Lewandowski and R. Mills contributed to the development of the magnetic resonance imaging protocols. J. Kitt, A. McCourt, H.R. Cutler, P.D. Sattwika, B. Raman, A.J. Lewandowski, and R. Mills contributed to magnetic resonance image acquisition and quality control. Magnetic resonance imaging analysis was performed by H.R. Cutler under the guidance of L.E.M. Finnigan and B. Raman. A. Estevez-Fernandez completed the interobserver reliability for the kidney volumes. Data collection and analysis were carried out by J. Kitt, A. McCourt, H. R. Cutler, P. D. Sattwika, Y. Kenworthy, C. Johnson, and S. Krasner. H.R. Cutler performed the statistical analysis. H.R. Cutler produced the tables and figures for the main paper and Supplemental Material. H. R. Cutler and P. Leeson wrote the manuscript. All authors contributed to the manuscript, revised, read, and accepted the final manuscript.

### Sources of Funding

The POP-HT study (Physician Optimized Postpartum Hypertension-Treatment Trial) was funded by a British Heart Foundation (BHF) Clinical Research Training Fellowship to J. Kitt (FS/19/7/34148) with additional support from the National Institute for Health and Care Research Oxford Biomedical Research Center and Oxford BHF Center for Research Excellence. The CAREFOL-HT study (Clinical Antenatal Randomised Study to Characterize Key Roles of Tetrahydrofolate in Hypertensive Pregnancies) and W. Lapidaire are funded by the Medical Research Council (MR/W003686/1). P. Leeson and C. Roman are supported by the Oxford National Institute for Health and Care Research Biomedical Research center. A.J. Lewandowski was supported by a BHF Intermediate Research Fellowship (FS/18/3/33292). The funders of this study had no role in study design, data collection, analysis, interpretation, or writing of the report. B. Raman is funded by a Wellcome Career Development Award fellowship (302210/Z/23/Z).

### Disclosures

None.

### Supplemental Material

Supplemental Methods

Figures S1–S6

## Supplementary Material


