# Supplementary material for: Subclinical Postpartum Renal Structure After Hypertensive Pregnancy Disorders
Source: Hypertension. 2025 Aug 31;82(11):1948–58. doi: 10.1161/HYPERTENSIONAHA.125.25130 (PMC12529983; doi:10.1161/HYPERTENSIONAHA.125.25130)
Supplement: Supplementary file 1 [file hyp-82-1948-s001.docx]

**Supplementary Material for the Subclinical Postpartum Renal Structure Following Hypertensive Pregnancy Disorders Magnetic Resonance Study**

INDEX OF SUPPLEMENTARY MATERIAL

1. Supplementary methods providing additional details of the inclusion and exclusion criteria, anthropometric measurements and magnetic resonance imaging acquisition protocols.
2. Figure S1: Interobserver variability limits of agreement, tested using Bland-Altman Analysis.
3. Figure S2: Intra-observer variability agreement. The difference in kidney volumes over multiple days, tested using Bland-Altman analysis.
4. Figure S3**:** Flowchart of the women involved in the study.
5. Figure S4: Linear regression models showing systolic (Panel A) and diastolic blood pressure 6-12 months postpartum (Panel B) vs total kidney volume indexed to body surface area.
6. Figure S5: Boxplots presenting total kidney volume indexed to body surface area for control vs intervention groups.
7. Figure S6: Boxplots displaying corticomedullary differentiation across control vs intervention groups.

**Supplementary Methods**
 **Inclusion Criteria for Hypertensive Cohort**
• Participant is willing and able to give informed consent for participation in the trial.
• Female, aged 18 years or above.
• Clinician confirmed diagnosis of either gestational hypertension or pre-eclampsia defined by the National Institute for Health and Care Excellence guidelines (see below).
• Requiring anti-hypertensive medication at the point of discharge from secondary care.
• Participant has clinically acceptable laboratory results and clinical course postpartum with no other adverse complicating factors requiring prolonged admission postpartum that would make participation unfeasible as judged by the chief investigator. Examples would include stroke sequalae, ongoing disseminated intravascular coagulation etc.
• In the investigator’s opinion, is able and willing to comply with all trial requirements including ownership of a ‘Smart-phone/Tablet’ and willing to use the smart-phone app if randomised to that arm.
• Sufficient competence in English Language to follow the app instructions and partake in the study, as judged by the chief investigator.

**Diagnosis Criteria**
Gestational hypertension diagnosis requires a blood pressure >140 mmHg systolic or > 90 mnHg diastolic on > 2 occasions after 20 weeks of this pregnancy. Preeclampsia diagnosis will be defined as per the National Institute for Health and Care Excellence guidelines and as follows: new onset of hypertension (>140 mmHg systolic or over 90 mmHg diastolic) after 20 weeks of pregnancy and the coexistence of one or more of the following new onset conditions:
• Proteinuria (spot urine protein/creatinine over 30 mg/mmol [0.3 mg/mg] or over 300 mg/day or at least 1 g/L [‘2 +’] on dipstick testing) or other maternal organ dysfunction:

- Renal insufficiency (creatinine 90 umol/L or more, 1.02 mg/dL);
- Liver involvement (elevated transaminases [ALT or AST over 40 IU/L] with or without right upper quadrant or epigastric abdominal pain);
- Neurological complications such as eclampsia, altered mental status, blindness, stroke, clonus, severe headaches or persistent visual scotomata;
- Haematological complications such as thrombocytopenia (platelet count below 150,000 cells/µL), disseminated intravascular coagulation or haemolysis;
- Utero-placental dysfunction such as foetal growth restriction, abnormal umbilical artery Doppler waveform analysis, or stillbirth.

**Exclusion Criteria for Hypertensive Cohort:**
• Significant renal or hepatic impairment that would affect safe medication titration and adjustment as part of the trial, as deemed by the chief investigator.
• Scheduled elective surgery (excluding caesarean sections) or other procedures requiring general anaesthesia during the trial.
• Participant with life expectancy of less than six months.
• Any other significant disease or disorder, which, in the opinion of the investigator, may either, put the participants at risk because of participation in the trial, or may influence the result of the trial, or the participant’s ability to participate in the trial.
• Participants who have participated in another research trial involving an investigational product in the past 12 weeks.
• An absolute contraindication to magnetic resonance imaging (as per magnetic resonance imaging safety questionnaire) precludes the women from having an magnetic resonance imaging but they can still participate in the remainder of the study
• Women with pre-existing hypertension will be excluded, as this is a separate pathology that would affect the efficacy of the study intervention and affect the primary and secondary outcomes of the study.

**Inclusion Criteria for Normotensive Cohort**

- Participant is willing and able to give informed consent for participation in the study
- Age >18 and ≤ 45 years
- Normotensive, blood pressures <140/90 mmHg throughout the antenatal period
- Less than two moderate risk factors for hypertensive disease in pregnancy according to the National Institute for Health and Care Excellence guidelines for management of hypertension in pregnancy
- SFlt/PIGF ratio < 35

**Exclusion Criteria for Normotensive Cohort**

- Diagnosis of hypertensive disorder of pregnancy
- Use of beta blockers such as atenolol or equivalent
- History of cardiac impairments including uncontrolled arrhythmia, unstable angina, decompensated congestive heart failure or valve disease
- History of pre-existing chronic renal disease
- Contraindication to magnetic resonance imaging e.g. pregnancy, pacemaker, ferromagnetic implant, shrapnel injury, severe claustrophobia
- Any known trisomy, foetus with congenital heart defect, foetus at a high risk of heart disease, known infection of foetus or known severe anaemia

**Anthropometry measurements**Height and weight were measured, after removal of shoes and heaving clothing, to the nearest centimetre and 0.1kg, respectively. Height was measured on a free-standing stadiometer and weight was measured on scales. Body mass index (BMI) was calculated using the formula: BMI (kg/m^2^) = weight (kg) / height (m)^2^. Hip circumference was measured at the widest part of the gluteal region, whilst waist circumference was measured just above the belly button. Waist to hip ratio was then calculated. Left arm circumference was measured at the widest part of the upper arm while relaxed.

**Magnetic Resonance Imaging Acquisition**
Volumetric images were acquired using a slice thickness of 3.0 mm, 320 × 256 matrix, 380 mm field of view, 1.2×1.2×3 mm acquired voxel size, 1.26 ms echo time, 3.17 ms repetition time and 7° flip angle. T1 maps were acquired with a 5-3s - 3 sampling scheme 40 [oblique coronal; repetition time/echo time = 2.7/1.15 ms; R=4; flip angle =35°; 320 × 320 mm^2^ field of view, 194 × 192 matrix, 5.5 mm slice thickness, simulated heart rate = 1s, TI1 =100ms, TI2 = 180 ms].

**
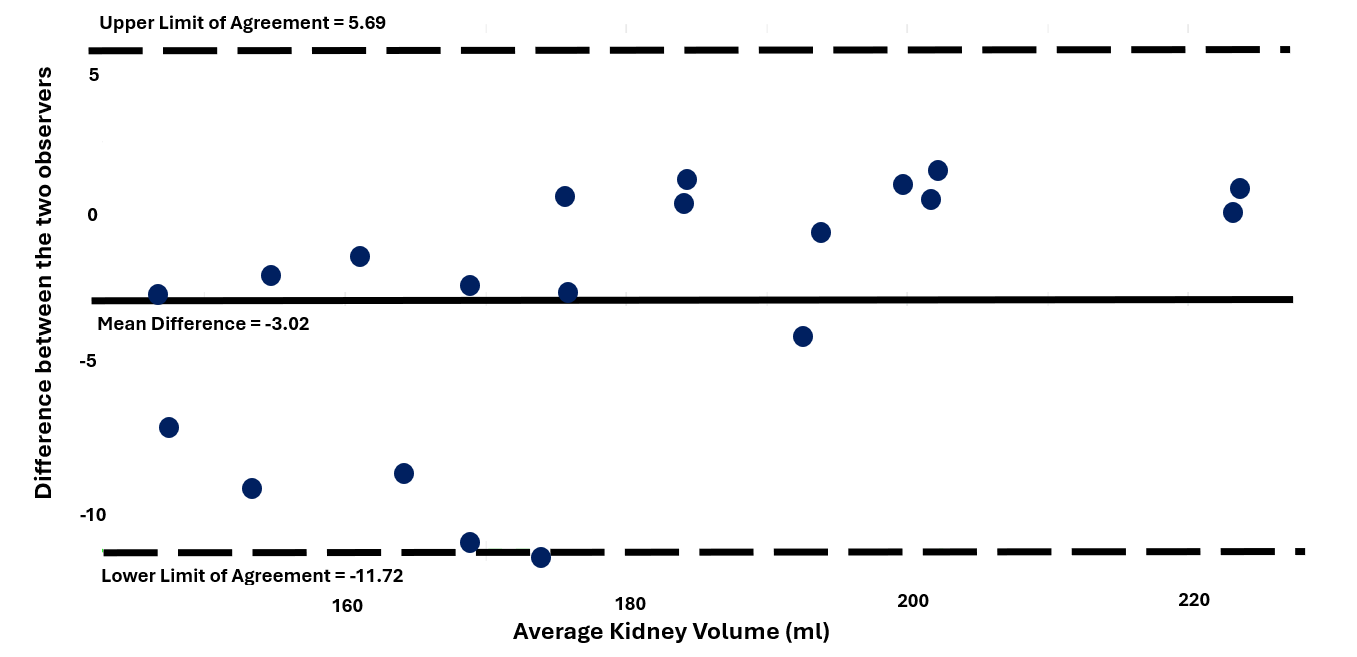
**

**S1.** Interobserver variability limits of agreement, tested using Bland-Altman Analysis.

**
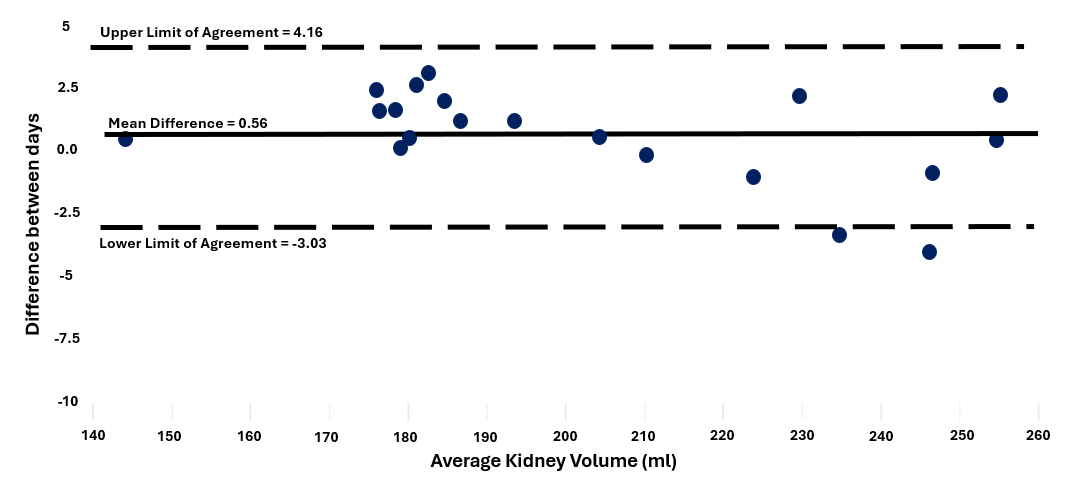
**

**S2.** Intra-observer variability agreement. The difference in kidney volumes over multiple days, tested using Bland-Altman analysis.


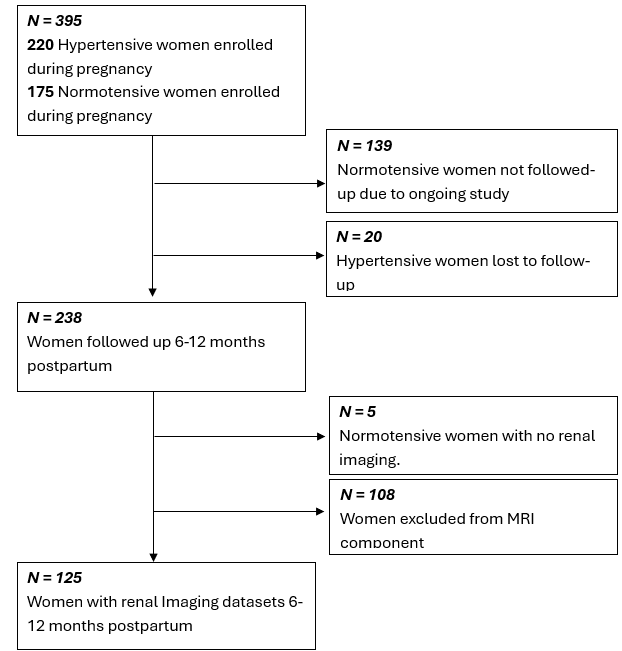


**S3.** Flowchart of the women involved in the study.


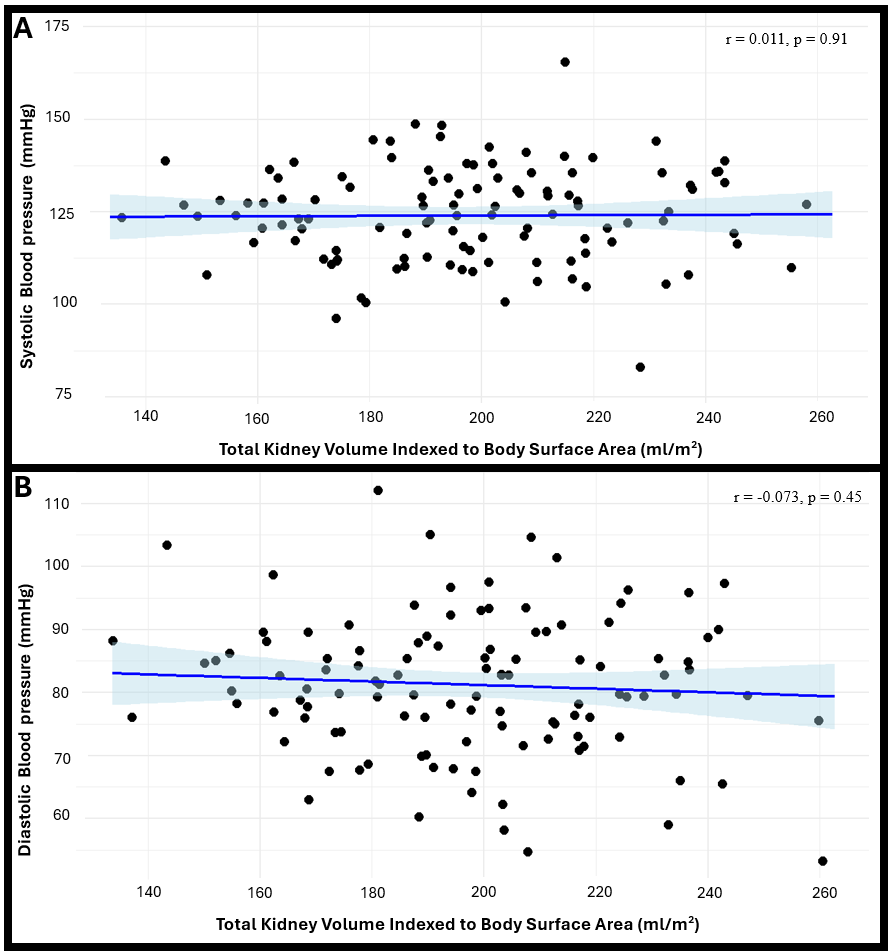


**S4.** Linear regression models showing systolic **(Panel A)** and diastolic blood pressure 6-12 months postpartum **(Panel B)** vs total kidney volumes indexed to body surface area.

**
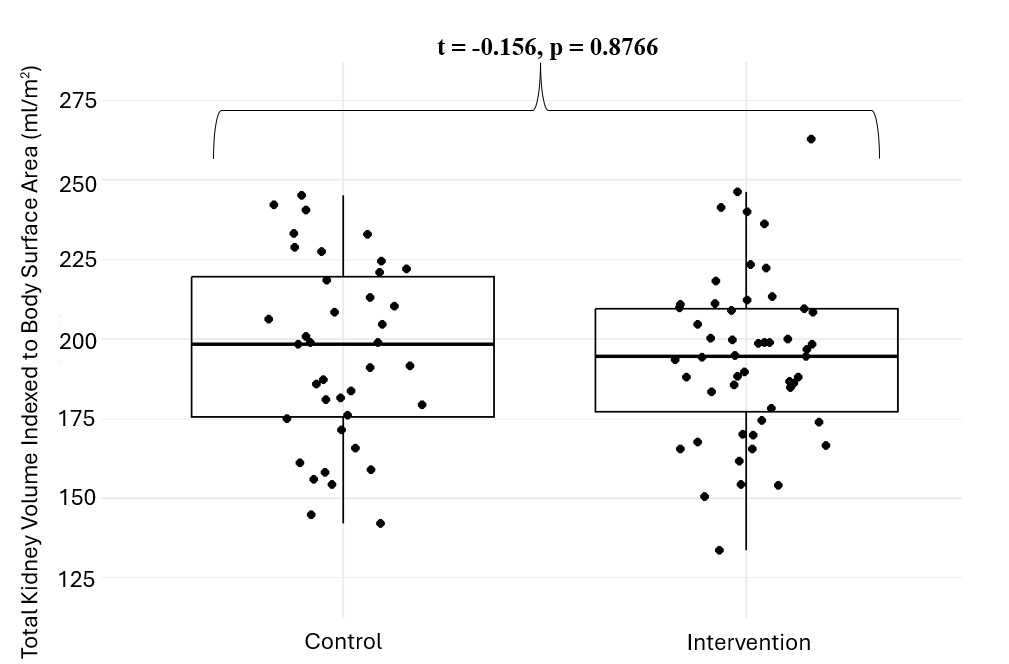
**

**S5.** Boxplots presenting total kidney volume indexed to body surface area for control vs intervention groups.

**
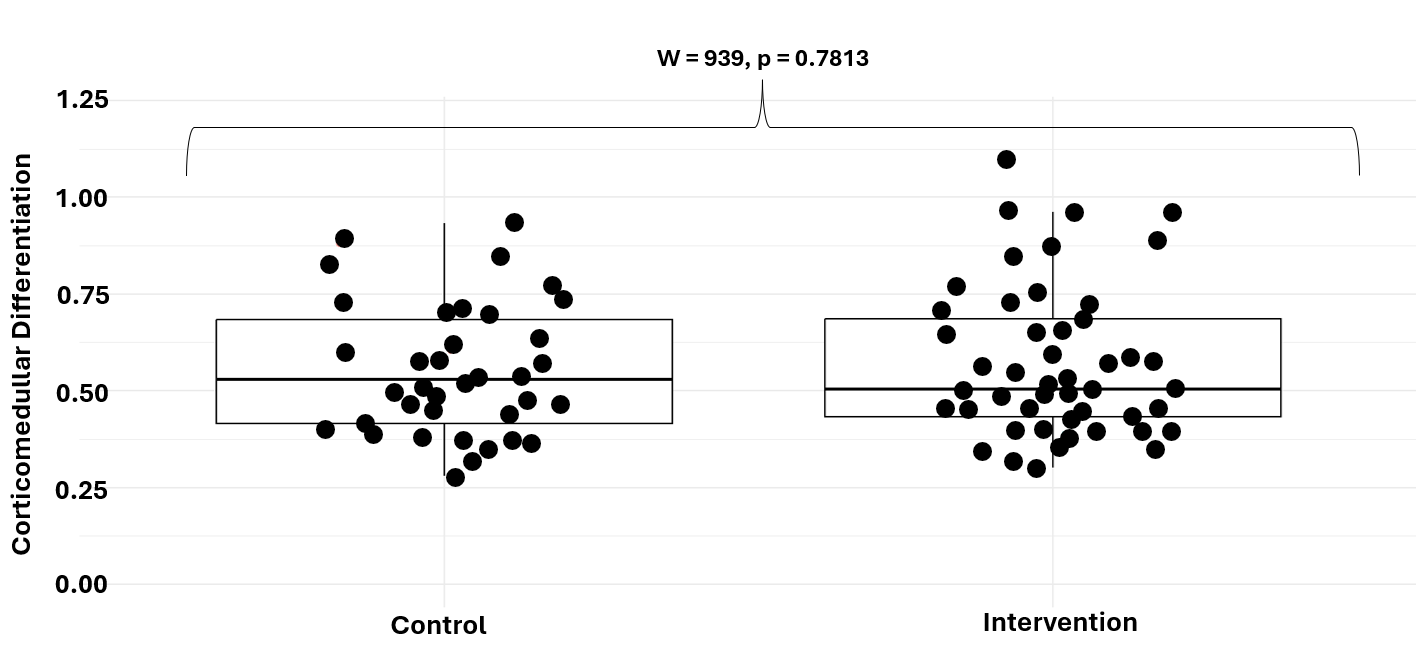
**

**S6.** Boxplots displaying corticomedullary differentiation across control vs intervention groups.
