# Supplementary material for: Subclinical Postpartum Renal Structure After Hypertensive Pregnancy Disorders
Source: Hypertension. 2025 Aug 31;82(11):1948–58. doi: 10.1161/HYPERTENSIONAHA.125.25130 (PMC12529983; doi:10.1161/HYPERTENSIONAHA.125.25130)
Supplement: Supplementary file 2 [file hyp-82-1948-s002.pdf]

*** CONSORT Reporting Guidelines**

This study is reporting on a clinical randomized control trial:

Yes

Clinical trial reports should comply with the Consolidated Standards of Reporting Trials (CONSORT) including its additional extensions as appropriate. This should include a flow diagram presenting the screening, enrollment, intervention allocation, follow-up, and data analysis with number of subjects for each. Please refer specifically to the CONSORT Checklist of items to include when reporting a randomized clinical trial and provide the completed checklist at the time of submission.

Please confirm and provide the following details at this time:

Clinical Trial Registration

Clinical trials that are submitted for publication must be registered in an appropriate online trial registry at or before the onset of participant enrollment. This requirement applies to all clinical trials, including Phase 1 studies. Any research study that prospectively assigns human participants or groups of humans to one or more health-related intervention(s) to evaluate the effects on health outcomes is considered a clinical trial.

Was there randomization or prospective assignment of human subjects to an intervention or comparison group to study the relation between a health-related intervention and a health outcome?

Yes

Is this study registered on a publicly accessible web site?

Yes

If yes, please provide:
Complete name of the trial registry

POP-HT

Registry's URL:

clinicaltrials.gov

Trial registration number:

NCT04273854

In accordance with the Clinical Trial Registration Statement from the International Committee of Medical Journal Editors, all clinical trials in AHA/ASA journals must be registered in a public trials registry at or before the onset of participant enrollment.

Study registration date

20/02/2020

Study enrollment date

21/02/2020

Diverse Representation - Steering Committee

Were steps taken to ensure diverse representation among the trial steering committee, and if so, are these efforts outlined in the Methods including demographic information? If No, a lack of diversity should be explained and listed as a limitation.

Yes

For more information, please see Clinical Trial Registration: A Statement From the International Committee of Medical Journal Editors.

Sex and Race/Ethnicity Specific Results

Sex and race/ethnicity specific results of the trial's primary outcomes are reported regardless of whether there are significant differences by sex or race.

Yes

Diverse Representation - Participants

Were steps taken to ensure diverse representation among trial participants, and if so, are these efforts outlined in the methods? If No, a lack of diversity should be explained and listed as a limitation.

Yes

Date completed: 07/29/2025 07:04:22
User pid: 6154
